# Relation between metabolic syndrome and body compositions among Chinese adolescents and adults from a large-scale population survey

**DOI:** 10.1186/s12889-017-4238-3

**Published:** 2017-04-20

**Authors:** Tao Xu, Junting Liu, Junxiu Liu, Guangjin Zhu, Shaomei Han

**Affiliations:** 1 Institute of Basic Medical Sciences, Chinese Academy of Medical Sciences & School of Basic Medicine, Peking Union Medical College, Beijing, 100005 China; 2 Department of Epidemiology, Capital Institute of Pediatrics, Beijing, 100020 China; 3 Tufts Friedman School of Nutrition Science and Policy, 150 Harrison Ave, Boston, MA 02111 USA

**Keywords:** Metabolic syndrome, Body composition, Percentage of body fat, Body mass index, Waist-to-height ratio, Screening

## Abstract

**Background:**

Few nationally representative surveys regarding body composition and metabolic syndrome (MetS) have been done in a large-scale representative Chinese population to explore the prediction of body composition indicators for MetS. The objective of this study was to examine the relation of body composition and MetS and to determine the optimal cut-off values of body composition indicators that predict MetS in a large representative Chinese sample based on multiple provinces and ethnicities, covering a broad age range from 10 to 80 years old.

**Methods:**

The subjects came from a large-scale population survey on Chinese physiological constants and health conditions conducted in six provinces. 32,036 subjects completed all blood biochemical testing and body composition measure. Subjects meeting at least 3 of the following 5 criteria qualify as having MetS: elevated blood pressure, lower high density lipoprotein cholesterol level, higher triglyceride level, higher fasting glucose level and abdominal obesity.

**Results:**

The total prevalence rate of MetS for males (9.29%) was lower than for females (11.58%). The prevalence rates were 12.03% for male adults and 15.57% for female adults respectively. The risk of MetS increased 44.6% (OR = 1.446, 95%CI: 1.414–1.521) for males and 53.4% (OR = 1.534, 95%CI: 1.472–1.598) for females with each 5% increase of percentage of body fat. The risk of MetS increased two-fold (OR = 2.020, 95%CI: 1.920–2.125 for males; OR = 2.047, 95%CI: 1.954–2.144 for females respectively) with each 5% increase of waist-hip ratio. The risk of MetS increased three-fold (OR = 2.915, 95%CI: 2.742–3.099 for males; OR = 2.950, 95%CI: 2.784–3.127 for females respectively) with each 5% increase of Waist**-**to-Height Ratio (WHtR). Areas under the receiver operating curve (AUC) of most body composition indicators were larger than 0.70 and the sensitivities and the specificities of most cut-off values were larger than 0.65. AUCs of WHR and WHtR were the largest. The optimal cut-off values of WHtR were 0.51 for males and 0.53 for females.

**Conclusion:**

MetS has become a serious public health challenge in China. Body composition variables were closely related to MetS and they were reliable indicators in the screening of the presence of MetS.

## Background

As a result of economic growth and associated sociodemographic changes, the burden from infectious diseases has diminished in China, but changes in lifestyle and diet have led to an increase in life expectancy and an increased burden of cardiovascular disease, diabetes and other chronic diseases [1–3]. Metabolic syndrome (MetS) is characterized by a clustering of cardiovascular risk factors, including abdominal obesity, elevated blood pressure and blood glucose, and dyslipidemia. MetS has been found to be associated with an increased risk of diabetes and cardiovascular disease morbidity and mortality, resulting in an enormous economic burden to society [4–7]. In China, Feng Y surveyed the prevalence of MetS among a rural population of one Chinese province [8]. Gu D reported that the prevalence rates of MetS were 9.8% for males and 17.8% for females in Chinese adults aged 35–74 [9]. Even with all of this information, more evidence is needed regarding the prevalence rate of MetS among representative Chinese populations that cover a broader age range and that include more minorities and provinces.

Excess body fat has been seen to be related to MetS [10]. Visceral adiposity was associated with body mass index (BMI), was essential to assess cardiometabolic risk and may serve as a marker and target of therapy in cardiometabolic disease [11]. Djibo DA found that body adiposity index could distinguish ethnic differences in MetS confirmed by percentage of body fat (PBF) [12]. Zhu S reported PBF thresholds that corresponded to the risk of MetS based on traditional BMI cutoffs and provided equations for estimating the risk of MetS for any given percentage of body fat, sex, and race [13]. Liu P found that higher fat mass index (FMI) levels appeared to be independently and positively associated with the presence of MetS regardless of BMI and PBF by assessing the body composition in a Chinese population who participated in the annual health check-ups in one hospital [14]. However, few nationally representative surveys on the relationship between body composition and MetS have been done in a large-scale representative Chinese population to explore the prediction validity of body composition indicators for MetS.

The objective of this study was to examine the relationship between body composition and MetS and to determine the optimal cut-off values of body composition indicators to predict MetS in a large representative sample of Chinese adolescents and adults based on multiple provinces and ethnicities, covering a broad age range from 10 to 80 years old.

## Methods

### Sample and participants

This study sample was part of a large-scale population survey regarding Chinese physiological constants and health conditions conducted in 2007–2011. The study has been approved by the review board of Institute of Basic Medical Sciences, Chinese Academy of Medical Sciences. This survey was carried out in six provinces of China, including Heilongjiang province, Hunan province, Sichuan province, Yunnan province, Inner Mongolia autonomous region and Ningxia Hui autonomous region. The two-stage cluster sampling method was used to select eligible subjects in each province. Two or three cities were sampled based on their economical status and then several communities were randomly selected within each city. In these selected communities, all eligible people were referred to as our survey subjects. Eligible people included those aged 10–80 years old who weren’t suffering from severe chronic diseases and had not run a high fever in the past 15 days. After signing informed consent forms, all subjects came to the temporary physical examination centers voluntarily to take part in the survey. Among 82,336 eligible subjects, 34,903 people (42.4%) were selected randomly to complete blood biochemical testing. After excluding subjects missing important measures, such as biochemical variables and body composition indicators, 32,036 subjects had complete data on all survey scales, blood biochemical testing and body composition measures. The completion rate was 91.8%.

### Clinical laboratory tests

A team of experienced doctors, nurses, and technicians performed measurements of study parameters with standard laboratory methods. All procedures were performed following a 9–12 h overnight fast and all subjects were told to consume a bland diet before blood testing. Blood samples were drawn from the antecubital vein of the arm. Total cholesterol, triglycerides, high-density lipoprotein cholesterol and low-density lipoprotein cholesterol were measured with a Beckman AU Series Automatic Biochemical Analyzer (Japan), using Sekisui Medical (Japan) reagents. Fasting blood glucose, uric acid, creatine, and blood urea nitrogen were measured with the same instrument, using Beckman AU reagents.

### Body composition

Body composition was measured with the bioelectric impedance method (BIA). The Biodynamics BI-310 body composition analyzer was manufactured by American Biodynamics Corporation with high sensitivity and specificity. The same brand and model was used for all body composition analyzers and were adjusted every day before measuring in order to minimize measurement error. All researchers were trained based on the training manual. Subjects were to fast, refrain from strenuous exercise, at least 4 h before measuring and refrain from drinking 24 h before measuring. When measuring, subjects were in a supine position, hands were placed flatwise away from their body 15 cm, and feet were kept apart at least 15 cm.

Body composition indicators included percentage of body fat (PBF), body mass index (BMI), fat mass index (FMI) and fat-free mass index (FFMI), Waist**-**to-Hip Ratio (WHR) and Waist**-**to-Height Ratio (WHtR). PBF was the proportion of body fat weight in total body weight. BMI was defined as weight (kg) divided by squared height (m^2^). FMI was defined as fat body mass (kg) divided by squared height (m^2^). FFMI was defined as lean body mass (kg) divided by squared height (m^2^). WHR was defined as waist circumference divided by hip circumference. WHtR was defined as waist circumference divided by height. All mass indexes were measured to the nearest 0.1 kg.

### Blood pressure measurement

Blood pressure was measured in the morning after subjects had rested for 5 min in the seating position with her or his back supported, feet on the floor and right arm supported with cubit fossa at heart level. The appropriate cuff was chosen based on their arm circumference. Blood pressure was measured based on one clinical visit with OMRON HEM-7000 electronic sphygmomanometers (OMRON Health-Care, Kyoto, Japan).

### Definition of metabolic syndrome

According to the National Cholesterol Education Program Adult Treatment Panel III (NCEP ATP III), persons meeting at least 3 of the following 5 criteria qualify as having MetS: higher fasting glucose level, elevated blood pressure, lower high density lipoprotein cholesterol (HDL-C) level, higher triglyceride level and abdominal obesity [15, 16]. The definitions are shown below in detail.

For adults:

1. Elevated blood glucose: fasting blood glucose ≥110 mg/dL or diagnosed diabetes;
2. Elevated blood pressure (systolic blood pressure ≥ 130 mmHg and/or diastolic blood pressure ≥ 85 mmHg) or history of hypertension;
3. Low HDL-C: serum HDL-C concentration < 40 mg/dL for males and <50 mg/dL for females;
4. Elevated triglyceride: serum triglyceride concentration ≥ 150 mg/dL;
5. Abdominal obesity: a waist circumference of ≥102 cm for males and ≥88 cm for females, as measured to the nearest 0.1 cm at the level of the navel, using a flexible steel tape.

For adolescents:

1. Elevated blood glucose: fasting blood glucose ≥110 mg/dL or diagnosed diabetes; 2) Elevated blood pressure: elevated blood pressure (systolic blood pressure ≥ 90th percentile and/or diastolic blood pressure ≥ 90th percentile by age and sex) or history of hypertension;
2. Low HDL-C: serum HDL-C concentration < 40 mg/dL;
3. Elevated triglyceride: serum triglyceride concentration ≥ 110 mg/dL;
4. Abdominal obesity: a waist circumference ≥ 90th percentile by age and sex.
5. The 90th percentiles of waist circumference and blood pressure came from the age- and sex-specific reference data established by the survey among Chinese adolescents [17, 18].

### Statistical analysis

The database was constructed with EPI3.02 software, with the data input twice by two data managers to guarantee the accuracy and integration of the data. Statistical analysis was conducted with SAS9.2 software. Two-tailed *P* < 0.05 was defined as statistically significant. Continuous data were described with mean and standard deviation (SD) and compared with t test analysis of variance. Categorical data were described with number and percentage and compared with Chi-square test. Logistic regression model was used to estimate odds ratio (OR) and its 95% confidence interval (CI) for exploring the relation between body metabolic measures and MetS with sociodemographic factors as covariates. Receiver operating curve (ROC) analyses were used to determine optimal cutoff points for PBF, BMI, FMI, FFMI, WHR and WHtR in relation to the area under the curve (AUC), sensitivity and specificity in males and females. The values of PBF, BMI, FMI, FFMI, WHR and WHtR that resulted in maximizing the Youden index (sensitivity +specificity-1) were defined as optimal cutoff points.

## Results

The average age was 32.9 ± 19.5 years for 15,272 males and 33.3 ± 18.9 years for 16,764 females. The subjects came from dozens of ethnicities, including Han (19,564, 61.0%), Yi (2830, 9.0%), Miao (603, 2.0%), Mongolia (2026, 7.0%), Tibetan (1243, 4.0%), Korean (1514, 5.0%), Hui (2973, 10.0%), Tujia (816, 3.0%) and others (467, 2.0%).

Of all 32,036 subjects, 60.73% of all subjects (*N* = 19, 499) had at least one clinical feature of MetS (1 or more risk factors). 33.71%, 16.69%, 7.72%, 2.38% and 0.37% of all subjects had one, two, three, four and five risk factors respectively. Risk factors in order of prevalence were elevated blood pressure (37.52%), elevated triglyceride (22.53%), low HDL-C (21.18%), elevated blood glucose (10.63%) and abdominal obesity (9.86%). The most prevalent grouping of two risk factors was blood pressure and low HDL-C (7.10%). The most prevalent grouping of three risk factors was elevated blood pressure, elevated triglyceride and low HDL-C (3.83%).

All in all, 3354 subjects were diagnosed as MetS and the prevalence rate was 10.47%. The prevalence rate of MetS for males (9.29%) was lower than females (11.58%). Among 20, 862 adults (aged 18 years old or above), 2926 ones were diagnosed as MetS and the prevalence rate was 14.03%. The prevalence rates of MetS were 12.03% for male adults and 15.57% for female adults respectively. The prevalence and related factors of MetS as related to different demographic characteristics were shown in details in Table 1. With aging, the prevalence rates increased gradually. Among minorities, Tibetan subjects held the lowest prevalence rate of MetS and Korean subjects the highest one.

**Table 1:** Prevalence (%) of metabolic syndrome about demographic characteristics

Demographic characteristics	Total	One component	Two components	Three components	Four components	Five components	MetS	*P* ^*^
Age (years)								<0.0001
10–17	11,174	35.51	12.49	3.00	0.77	0.06	3.83	
18–29	5198	32.90	8.77	2.15	0.42	0.02	2.60	
30–39	3940	33.10	17.08	7.26	1.45	0.18	8.88	
40–49	4327	32.89	22.65	11.72	3.00	0.35	15.07	
50–59	3654	31.86	24.66	15.68	5.77	0.74	22.19	
60–69	2478	30.31	26.11	18.04	7.87	1.53	27.44	
70–80	1265	37.87	23.16	16.92	4.90	1.74	23.56	
Gender								<0.0001
Male	15,272	34.32	17.95	7.41	1.73	0.15	9.29	
Female	16,764	33.15	15.53	8.01	2.98	0.56	11.58	
Occupation								<0.0001
Physical laborer	11,270	33.35	20.91	12.15	4.21	0.79	17.15	
Mental laborer	20,766	33.90	14.40	5.32	1.39	0.13	6.84	
Ethnicity								<0.0001
Han	19,564	32.59	16.58	8.30	2.68	0.42	11.40	
Yi	2830	40.32	17.99	5.97	1.31	0.18	7.46	
Miao	603	30.18	13.60	5.47	1.82	0.00	7.30	
Mongolia	2026	31.34	15.89	7.65	1.78	0.25	9.67	
Tibetan	1243	34.35	11.34	3.30	1.05	0.24	4.59	
Korean	1514	33.55	17.70	8.85	2.91	0.33	12.09	
Hui	2973	37.50	19.58	7.97	2.62	0.50	11.10	
Tujia	816	33.46	15.81	6.13	1.23	0.12	7.48	
Others	467	30.41	14.78	6.64	2.14	0.00	8.78	
Smoker								<0.0001
No	26,223	33.58	15.83	7.17	2.31	0.39	9.87	
Yes	5813	34.30	20.54	10.22	2.68	0.28	13.18	
Alcohol drinker								<0.0001
No	26,371	33.68	15.65	7.17	2.32	0.39	9.88	
Yes	5665	33.84	21.50	10.27	2.67	0.26	13.20	

^*^ *P* values were derived from chi-square test for compare the difference of the prevalence rates of MetS among different demographic characteristics

Given that there were significant differences of body composition indicators and MetS between the two genders, analyses on the relationship between body composition indicators and MetS was conducted on males and females separately. Table 2 indicates the comparisons of body composition indicators between subjects with MetS and without MetS by genders. PBF of subjects with MetS was significantly higher than those without MetS (21.08% VS 14.58% for males; 30.08% VS 22.9% for females). WHtR of subjects with MetS was significantly higher than those without MetS (0.54 VS 0.46 for males; 0.56 VS 0.46 for females). In addition, subjects without MetS had higher BMI, FMI, FFMI and WHR.

**Table 2:** Comparisons of body composition indicators between MetS and Non-MetS

	Male			Female		
	MetS	Non-MetS	*P* ^*^	MetS	Non-MetS	*P* ^*^
PBF (%)	21.08 ± 7.23	14.58 ± 8.32	<0.0001	30.08 ± 6.67	22.99 ± 7.53	<0.0001
BMI (kg/m^2^)	27.00 ± 3.60	21.74 ± 3.77	<0.0001	26.18 ± 3.59	21.38 ± 3.30	<0.0001
FMI (kg/m^2^)	21.17 ± 2.57	18.41 ± 2.79	<0.0001	18.17 ± 2.14	16.32 ± 2.02	<0.0001
FFMI (kg/m^2^)	5.81 ± 2.36	3.32 ± 2.18	<0.0001	8.00 ± 2.48	5.06 ± 2.17	<0.0001
WHR	0.92 ± 0.06	0.84 ± 0.07	<0.0001	0.89 ± 0.07	0.80 ± 0.06	<0.0001
WHtR	0.54 ± 0.06	0.46 ± 0.06	<0.0001	0.56 ± 0.06	0.46 ± 0.06	<0.0001

* t-test

Table 3 shows the ORs and their 95%CI of body composition indicators predicting MetS with various logistic regression models. The similar trend was found regarding the relationship between body composition indicators and MetS in two genders and two models. We found the risk of MetS would increase 44.6% (OR = 1.446, 95%CI: 1.414–1.521) for males and 53.4% (OR = 1.534, 95%CI: 1.472–1.598) for females with each 5% increase of PBF adjusting for age, gender, ethnicity, occupation, smoking and drinking with multivariate logistic models. Adjusting for the same demographic characteristics, the risk of MetS would increase 40.7% (OR = 1.407, 95%CI: 1.380–1.434) for males and 38.9% (OR = 1.389, 95%CI: 1.364–1.415) for females with each 1 kg/m^2^ increase of BMI. Meanwhile, the risk of MetS increased two-fold (OR = 2.020, 95%CI: 1.920–2.125 for males; OR = 2.047, 95%CI: 1.954–2.144 for females respectively) with each 5% increase of WHR. The risk of MetS increased three-fold (OR = 2.915, 95%CI: 2.742–3.099 for males; OR = 2.950, 95%CI: 2.784–3.127 for females respectively) that of non-MetS with each 5% increase of WHtR. ORs and their 95CI of other body compositions were shown in Table 3.

**Table 3:** ORs and 95% CI of body composition indicators for MetS

	Male				Female			
	Univariate		Adjusted^a^		Univariate		Adjusted^a^	
	OR	95% CI	OR	95% CI	OR	95% CI	OR	95% CI
PBF (5%)^b^	1.516	1.468–1.565	1.466	1.414–1.521	1.878	1.812–1.947	1.534	1.472–1.598
BMI (kg/m^2^)	1.414	1.390–1.439	1.407	1.380–1.434	1.454	1.430–1.478	1.389	1.364–1.415
FMI (kg/m^2^)	1.459	1.426–1.493	1.438	1.401–1.476	1.550	1.512–1.590	1.502	1.459–1.546
FFMI (kg/m^2^)	1.508	1.472–1.544	1.464	1.426–1.503	1.642	1.605–1.679	1.488	1.450–1.527
WHR (5%)	2.211	2.112–2.315	2.020	1.920–2.125	2.509	2.406–2.617	2.047	1.954–2.144
WHtR (5%)	2.891	2.741–3.049	2.915	2.742–3.099	3.264	3.110–3.426	2.950	2.784–3.127

^a^Adjusting for age, gender, ethnicity, occupation, smoking and drinking with multivariate logistic models

^b^ORs of PBF, WHR and WHtR were the ORs of 5% of PBF, WHR and WHtR

The areas under ROC curve, the cutoff values, and the most appropriate sensitivities and specificities of the indicators are presented separately for adults in Table 4 and adolescents in Table 5. As shown in Tables 4 and 5, the AUCs of most body composition indicators were larger than 0.70 and the sensitivities and the specificities of most cut-off values were larger than 0.65. For adults, the AUC of WHR (0.809 for males and 0.818 for females) and the AUC of WHtR (0.824 for males and 0.759 for females) were larger than those of other body composition indicators. The optimal cut-off values of WHR were 0.89 for males and 0.84 for females. The optimal cut-off values of WHtR were 0.51 for males and 0.53 for females. The optimal cut-off value of PBF for males was 17.78% which was smaller by about 10% than for females (27.45%). For adolescents, the AUC of BMI (0.717 for boys and 0.704 for girls) and the AUC of WHtR (0.771 for boys and 0.713 for girls) were larger than those of other body composition indicators. The optimal cut-off values of BMI were 21.95 kg/m^2^ for boys and 21.11 kg/m^2^ for girls. The optimal cut-off values of WHtR were 0.46 for both boys and girls. The optimal cut-off value of PBF for boys was 14.56%, which was smaller by about 8% than for girls (22.36%).

**Table 4:** Sensitivity, specificity and AUC in prediction of MetS for adults

Indicators	Cut-off value	Sensitivity	Specificity	AUC	95% CI
PBF (%)					
Male	17.78	0.712	0.579	0.695	0.681–0.709
Female	27.45	0.747	0.649	0.745	0.733–0.757
BMI (kg/m^2^)					
Male	24.77	0.799	0.670	0.809	0.797–0.821
Female	24.33	0.760	0.743	0.820	0.809–0.830
FMI (kg/m^2^)					
Male	20.38	0.683	0.661	0.730	0.715–0.745
Female	17.62	0.643	0.682	0.714	0.701–0.728
FFMI (kg/m^2^)					
Male	4.46	0.739	0.622	0.747	0.734–0.761
Female	6.91	0.727	0.745	0.799	0.788–0.810
WHR					
Male	0.89	0.809	0.620	0.781	0.769–0.794
Female	0.84	0.818	0.320	0.823	0.812–0.833
WHtR					
Male	0.51	0.824	0.643	0.807	0.795–0.819
Female	0.53	0.759	0.798	0.856	0.847–0.866

**Table 5:** Sensitivity, specificity and AUC in prediction of MetS for adolescents

Indicators	Cut-off value	Sensitivity	Specificity	AUC	95% CI
PBF (%)					
Male	14.56	0.678	0.710	0.732	0.695–0.769
Female	22.36	0.637	0.773	0.697	0.660–0.734
BMI (kg/m^2^)					
Male	21.95	0.717	0.870	0.857	0.827–0.887
Female	21.11	0.704	0.759	0.787	0.754–0.820
FMI (kg/m^2^)					
Male	18.72	0.634	0.791	0.773	0.739–0.807
Female	16.67	0.610	0.741	0.721	0.685–0.757
FFMI (kg/m^2^)					
Male	3.50	0.663	0.815	0.782	0.745–0.819
Female	4.65	0.695	0.703	0.749	0.713–0.785
WHR					
Male	0.85	0.698	0.769	0.791	0.759–0.823
Female	0.80	0.671	0.687	0.730	0.697–0.767
WHtR					
Male	0.46	0.771	0.815	0.843	0.812–0.874
Female	0.46	0.713	0.796	0.813	0.783–0.844

## Discussions

MetS is associated with the development of diabetes, cardiovascular diseases, and kidney diseases, which are the leading cause of mortality worldwide [19–22]. In the present study, we found that the prevalence rate of MetS was 9.29% for males and 11.58% for females. Among adults (aged 18 years old or above), the prevalence rate of MetS was 12.03% for male and 15.57% for female respectively. As overweight and obesity increased worldwide, MetS itself will also become increasingly common. Without a national emphasis on the prevention and control of the metabolic syndrome, the burden of this problem in China is likely to increase in the near future.

To the best of our knowledge, this is the first study that examined the relationship between body composition variables and MetS as well as determined the optimal cut-off values of body composition variables that predict metabolic syndrome with a large representative Chinese sample including six provinces and many minority groups, covering a broad age range from 10 to 80 years old. Our survey was conducted with standard protocols and instruments and strict training processes and vigorous quality assurance programs were used to ensure the quality of the data collection.

In this study, PBF, BMI, FMI, FFMI, WHR and WHtR were found be closely related with MetS. The AUCs of most body composition indicators were larger than 0.70 and the sensitivities and the specificities of most cut-off values were larger than 0.65, which showed good predictability of body composition indicators for MetS.

We found the optimal cut-off values of BMI predicting MetS (24.77 kg/m^2^ for adults males and 24.33 kg/m^2^ for adults females) were almost same as the standard overweight cutoff (24 kg/m^2^) for Chinese adults [23]. The optimal cut-off values of WHtR were 0.51 for male adults and 0.53 for adults females, which indicated that a Chinese person may be more susceptible to suffer from MetS when his/her waist circumference is greater than half of his/her height. A previous meta-analysis showed that a reduction of overweight (BMI to <24 kg/m^2^) might reduce the incidence of stroke by 15% in men and 22% in women in China [24]. Another study also suggested that BMI, waist circumference and WHtR values were all associated with metabolic risk factors, and they may equally predict multiple metabolic risk factors [25]. A Korean study showed that WHtR was a better screening tool than BMI and waist circumference for adult metabolic risk factors was the best screening tool for evaluation MetS in Korean men [26, 27].

The optimal cut-off values of FMI for males were larger by about 2.6 kg/m^2^ for adults and 2.0 kg/m^2^ for adolescents than those for females. The optimal cut-off values of FFMI for males were smaller by about 2.5 kg/m^2^ among adults and 1.1 kg/m^2^ among adolescents than those for females. The optimal cut-off value of PBF for male adults was 17.78%, which was smaller by about 10% than for female adults (27.45%). The optimal cut-off value of PBF for boys was 14.56% which was smaller by about 8% than for girls (22.36%). This may be ascribed to the different influence of body fat on the two genders. Body fat mainly inhabits internal organs for males and under subcutaneous tissues for females, which is called ectopic fat disposition [28]. Body fat in internal organs plays a role in impairing insulin resistance [29, 30]. Visceral adiposity was more strongly associated with incident MetS than subcutaneous fat [11]. Therefore, the cut-off values of PBF and FFMI for females were larger than for males. In addition, females had a much higher prevalence of the MetS than did males. This difference might be due to a higher prevalence of abdominal obesity and low HDL-cholesterol in women compared with men [9].

Several limitations have to be mentioned. First, due to the cross-sectional design, it is not possible to explore the causal relationship between body composition and MetS. Second, given that our analysis excluded subjects suffering from some severe chronic diseases and having fever in the last 15 days, we believe the true prevalence of MetS in Chinese population would be underestimated. Third, subjects in this study did not include children aged <10 years old and elders aged >80 years old, which should be studied further in future.

## Conclusions

Despite these limitations, our findings showed that MetS has become a serious public health challenge in China. Body composition variables PBF, BMI, FMI, FFMI, WHR and WHtR were closely related with MetS and they were reliable indicators in the screening of the presence of MetS.

## Abbreviations

AUC: Area under the curve
BMI: Body mass index
CI: Confidence interval
DBP: Diastolic blood pressure
FFMI: Fat-free mass index
FMI: Fat mass index
HDL-C: High density lipoprotein cholesterol
MetS: Metabolic syndrome
NCEP ATP III: National Cholesterol Education Program Adult Treatment Panel III
OR: Odds ratio
PBF: Proportion of body fat weight
ROC: Receiver operating curve
SBP: Systolic blood pressure
SD: Standard deviation
WC: Waist circumference
WGOC: Working Group on Obesity in China
WHR: Waist**-**to-Hip Ratio
WHtR: Waist**-**to-Height Ratio
